# Faith in Fat: A Multisite Examination of University Students’ Perceptions of Fat in the Diet

**DOI:** 10.3390/nu12092560

**Published:** 2020-08-24

**Authors:** Matthew J. Landry, Jasmine M. Olvany, Megan P. Mueller, Tiffany Chen, Dana Ikeda, Danielle Sinclair, Lesley E. Schatz, Priscilla Connors, Robert T. Valgenti, Ghislaine Amsler Challamel, Christopher D. Gardner, Peggy Policastro

**Affiliations:** 1 Stanford Prevention Research Center, Stanford University, Palo Alto, CA 94305, USA; cgardner@stanford.edu; 2 Department of Genetics and Genome Sciences, Case Western Reserve University, Cleveland, OH 44106, USA; jmo85@case.edu; 3 Department of Food Science and Human Nutrition, Colorado State University, Fort Collins, CO 80523, USA; megan.mueller@colostate.edu; 4 New Jersey Institute for Food, Nutrition, and Health/Dining Services, Rutgers University, New Brunswick, NJ 08901, USA; tc687@scarletmail.rutgers.edu (T.C.); dmi42@scarletmail.rutgers.edu (D.I.); peggyp@rutgers.edu (P.P.); 5 Campus Dining with Housing Dining & Auxiliary Enterprises, University of California, Santa Barbara, CA 93106, USA; dsinclair@ucsb.edu; 6 NC State Dining, North Carolina State University, Raleigh, NC 27695, USA; lestewa2@ncsu.edu; 7 Department of Hospitality & Tourism Management, University of North Texas, Denton, TX 76203, USA; priscilla.connors@unt.edu; 8 Department of Religion and Philosophy, Lebanon Valley College, Annville, PA 17003, USA; valgenti@lvc.edu; 9 Menus of Change University Research Collaborative, Stanford, CA 94305, USA; gchallamel@stanford.edu

**Keywords:** dietary fat, dietary recommendations, nutrition knowledge, consumer behaviors

## Abstract

Despite recent relaxation of restrictions on dietary fat consumption in dietary guidelines, there remains a collective “fear of fat”. This study examined college students’ perceptions of health among foods with no fat relative to foods with different types of fats (unsaturated and saturated). Utilizing a multisite approach, this study collected data from college students at six university dining halls throughout the United States. Data were available on 533 students. Participants were 52% male and consisted largely of first-year students (43%). Across three meal types, the no-fat preparation option was chosen 73% of the time, the unsaturated fat option was selected 23% of the time, and the saturated fat option was chosen 4% of the time. Students chose the no-fat option for all meal types 44% of the time. Findings suggest that college students lack knowledge regarding the vital role played by the type and amount of fats within a healthy diet. Nutrition education and food system reforms are needed to help consumers understand that type of fat is more important than total amount of fat. Efforts across various sectors can encourage incorporating, rather than avoiding, fats within healthy dietary patterns.

## 1. Introduction

Dietary fat intake is an essential facet of a healthy diet for cell functioning and for facilitating the utilization of fat-soluble vitamins and the bioavailability of carotenoids [1,2]. It also contributes to the texture, flavor, and palatability of foods and may increase satiety as fats can take longer to digest compared with other macronutrients [3,4]. Nutritional guidelines on dietary fats have changed in recent decades [5]. In the 1970s, 80s, and 90s, the focus was on limiting fat and choosing low-fat options [6]. Concurrently, food manufacturers heavily marketed low-fat items such as low-fat snacks and breakfast cereals. These low-fat products had calorie levels per serving that were similar to the higher-fat alternatives, but were higher in added sugars and refined grains [7]. The 2015–2020 Dietary Guidelines for Americans recommends foods higher in mono- and polyunsaturated fats which are proven to be healthier than saturated fats [8]. The Scientific Report of the 2020 Dietary Guidelines Advisory Committee carried forward the recommendation to replace saturated fats with polyunsaturated fats [9], although there is some disagreement about these recommendations among researchers [10]. Despite these guidelines, low-fat options are still heavily marketed, and research suggests that most adults do not know which fats are healthy and have a limited knowledge of how dietary fats differ among food sources [11,12,13,14,15,16]. This discrepancy between scientific knowledge and consumer beliefs perpetuates less healthy food choice behaviors, such as avoiding or limiting dietary fat consumption altogether.

Few studies have explicitly evaluated the knowledge and perceptions of dietary fats among college students. This is an important demographic group, as eating behaviors adopted in young adulthood often persist for decades [17,18]. Contemporary college students are among the first generation to have likely received education on healthy fats in their nutrition education curriculum in elementary and/or middle school [19]. However, their parents/guardians would have grown up when advertisement around the benefits of low-fat diets was prevalent, which may have impacted messaging they received at home. Multiple studies have addressed current perceptions of dietary fat, but they all focused intensely on either low-fat diets or trans-fats. Davy et al. (2006) and Steptoe et al. (2002) found that most college students view low-fat diets as being important for weight loss and heart disease [20,21]. In a study by Josti and Kovacs (2010), almost three-quarters of students reported knowing that trans-fat is unhealthy [22]. Qualitative research also suggests that some students view low-fat versions of foods as being healthier compared to regular versions of those foods items, while others are aware that the type of fat matters for health [23,24]. Currently, there is a paucity of empirical studies that address college students’ perceptions about the healthfulness of no-fat foods versus foods with different types of fat.

Given the importance of the type of dietary fat intake for health and the declines in diet quality that occur during the transition into college [25,26], it is important to understand college students’ knowledge and perceptions of healthy dietary fats. The concept of “go good fat not low fat” is a central principle of the Menus of Change initiative [27], created by the Culinary Institute of America and Harvard T. H. Chan School of Public Health, with a mission to improve the American diet by influencing all facets of the culinary profession. A complete list of Menus of Change principles is provided in Supplementary Figure S1. One particular arm of this initiative, called the Menus of Change University Research Collaborative (MCURC) [28], includes an international network of partners from over 60 university and college campuses that uses campus dining halls as living laboratories for behavior change interventions based on Menus of Change principles. Utilizing this unique collaboration of institutions, this study aims to fill an important gap in the literature by examining how college students in the United States perceive the relative healthfulness of no-fat foods relative to foods with different types of fats (unsaturated and saturated).

## 2. Materials and Methods

The ‘Faith in Fat’ study (protocol number: E12-486; review board: Rutgers University) focused on students’ knowledge and perceptions of dietary fat. Inclusion criteria for participating schools included being part of the MCURC research working group, having at least one dining hall with a main serving area, having space to set up an information table, and having undergraduate volunteers willing to carry out study protocols at each site. All 29 member schools that met these criteria were invited to participate. Eight schools expressed initial interest in participating in the study. Following information sessions about the study, six schools participated in data collection. Schools included are diverse in geographic location within the United States, size (undergraduate population ranging from 1700 to over 33,000), urbanicity, and both private and public institutions. A standardized protocol was used across all MCURC institutions and was executed by undergraduate volunteers. Characteristics of schools participating in the Faith in Fat study are provided in Supplementary Table S1.

### 2.1. Faith in Fat Study Description

Participants were derived from a convenience sample of college students in campus dining halls across six MCURC institutions. College students were surveyed to assess which food preparation method they perceived to be the healthiest. A 3-food item (salad (dressing), protein (chicken), dessert (cookie)) × 3-fat option (no fat, unsaturated fat, saturated fat) study design was used. Each of the three food items differed in the type of fat described in the preparation method (no fat, primarily unsaturated fat, primarily saturated fat) of the food (Table 1). The foods used in this experiment were chosen because they are regularly available in campus dining halls and represent three major categories of dining options: appetizer, entree, and dessert.

During one to four lunch or dinner meal periods selected by each MCURC study team of each participating universities in Spring 2018 asked student diners to voluntarily participate in the study. Those who agreed were directed to a nutrition tabling event in the campus dining halls and were presented with a tri-fold board displaying three preparation methods with various fat options for each food item. Trained research assistants individually prompted each volunteer at the nutrition booth to choose which food preparation they believed to be the healthiest for each food item. Researchers recorded the students’ responses for each food item on a data sheet and noted the corresponding sex and academic year of the participant. No other identifiable information was collected; all responses remained anonymous. After completing the activity, researchers provided each participant, regardless of response, with a small prize (e.g., small erasers or a snack-portion bag of nuts).

### 2.2. Ethics

Verbal informed consent was obtained from each student. These studies were conducted according to the guidelines laid down in the Declaration of Helsinki and all procedures involving human subjects were approved by the Institutional Review Board (IRB) of Rutgers University. Each school’s principal investigator signed a letter of cooperation to conduct study related activities at their school in accordance with the study protocol approved by their individual institutional IRB.

### 2.3. Statistical Analysis

Basic summary statistics (mean, percentage) were used to describe the characteristics of students. Frequencies were used to determine whether college students perceive no-fat foods as more health-promoting than foods with primarily unsaturated fat or primarily saturated fat. Chi-square (χ2) tests were used to determine differences between participant characteristics and meal type or food item. Data were analyzed in RStudio (Version 1.2.5042, RStudio Team, 2020). After basic summary statistics, each fat option in the Faith in Fat study was coded into a binary outcome variable by marking the unsaturated fat response as correct and other responses (no-fat or saturated fat) as incorrect. Utilizing this outcome, unadjusted logistic regression was run on each plate predicted by each of the collected variables. This allowed for an exploratory analysis of estimates of effect sizes and potential associations. Regression analyses were run in R (Version 3.6.2, R Core Team, 2019) For the purpose of eliminating small cells, the single participant who reported a non-binary sex from the sex variable was removed.

## 3. Results

Faith in Fat Results

Data were available from 533 students. Characteristics of students across the six MCURC schools that participated in the Faith in Fat study are presented in Table 2. Participants were 52% male and consisted largely of first-year students (43%).

**Figure 1:**
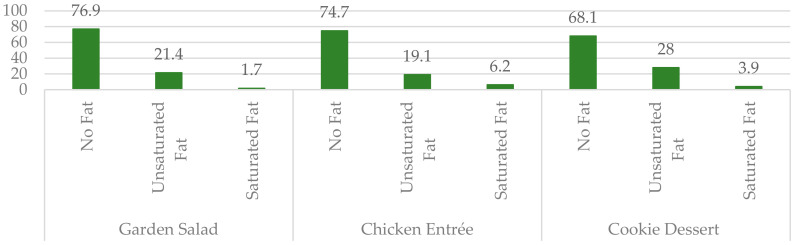
Frequency of fat option choice per meal type in the Faith in Fat study. Across all meal types, the no-fat preparation option was chosen 73% of the time.

Most participants chose the no-fat option for the garden salad (77%), chicken entrée (75%), and the cookie dessert (68%) (Figure 1). Across all meal types, the no-fat preparation option was chosen 73% of the time, the unsaturated fat option was chosen 23% of the time, and the saturated fat option was chosen 4% of the time. Forty-four percent of participants chose the no-fat option for all three meal types.

Although this study was not designed to detect changes between demographic characteristics, chi-square results of characteristics and meal fat options are provided in Table 3. There was a significant difference of responses by sex for the oatmeal cookie dessert, by year for the garden salad, and by school for the garden salad and chicken breast entrée. Frequencies of fat option choice by sex, school, and year classification are provided in Supplementary Tables S2–S4.

**Figure 2:**
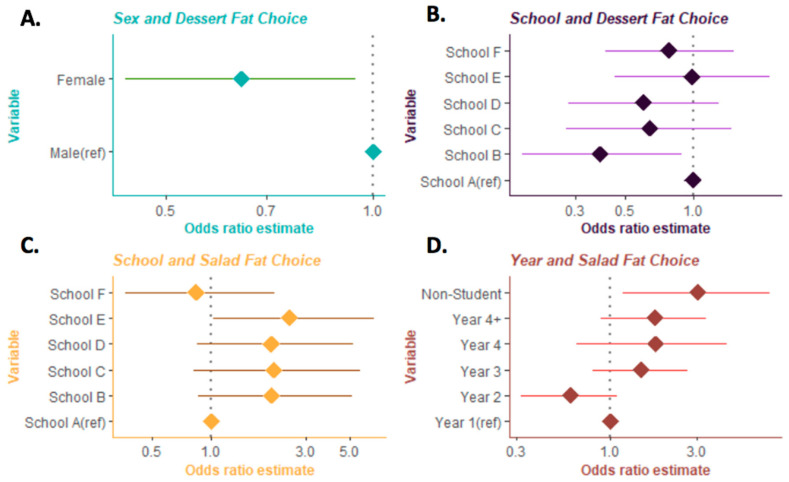
Estimating the odds ratio of variables with one or more associations. Each plate was transformed into a binary outcome based on unsaturated fat choice versus no-fat or saturated fat options. Plotted ORs of univariate logistic regression models that had at least one categorical level that associated with fat choice. (**A**) Females were less likely to choose the unsaturated fat dessert choice (*p* = 0.024). (**B**) School B were less likely to choose correct dessert fat (*p* = 0.026). (**C**) School E were more likely to choose the unsaturated fat salad fat than School A (*p* = 0.058). (**D**) Non-students were more likely to choose unsaturated fat salad than baseline (*p* = 0.018).

In exploratory analysis, females were significantly less likely to identify the unsaturated fat option as healthy compared to males OR = 0.64 (CI 95% = 0.44–0.94) (Figure 2A). There were several other categorical variables that had levels which associated but did not globally associate with the outcome dish. For dessert, we found that School B was significantly less likely to choose the unsaturated fat dessert than the School A reference OR = 0.39 (CI 95% = 0.17–0.90) (Figure 2B). School E was more likely to choose the unsaturated fat in the salad dish as compared to the School A reference OR = 3.07 (CI 95% = 1.19–7.70) (Figure 2C). The final marginal association we found was that non-students were also more likely than first years to choose the unsaturated fat option for the salad dish OR = 2.54 (CI 95% = 1.02–6.79) (Figure 2D). The entrée meal was the only outcome for which no marginal differences were detected. Overall, the collected variables (sex, school, and year classification) provided only limited additional resolution between individuals who recognize the benefit of healthy fats over no-fat options.

## 4. Discussion

The 2015–2020 Dietary Guidelines for Americans recommend that consumption of saturated fat should be limited to <10% of daily calories and should be replaced with unsaturated fat, particularly polyunsaturated fats [8]. In this study, college students perceived that non-fat options of three different meal types were healthier compared to an unsaturated fat option and a saturated fat option. The type of meal tested did not impact the perception of fat. This study provides empirical evidence of college students’ incorrect perceptions about the healthfulness of no-fat foods versus unsaturated and saturated fat foods.

The “fear of fat” began in the 1950s, with the emergence of evidence linking saturated fat with greater risk for cardiovascular disease [6]. As a result, low-fat, high-carbohydrate diets were recommended in the 1970s and onwards for weight loss and reducing cardiovascular disease risk. A public desire to avoid fat drove food industry reformulation of food products by food manufacturers and culinary menus marketing dishes that center around reduced or low fat. This had repercussions, as fat was most often replaced by refined carbohydrates and added sugars [7]. As fat consumption decreased, carbohydrate intake increased and was accompanied by significant increases in total energy and obesity rates in the United States [29,30,31]. Evidence has accumulated that supports dietary guidance more specifically focusing on the type of fat rather than total amount of fat. Recommendations in the 2015–2020 Dietary Guidelines for Americans [32], the recent Scientific Report of the 2020 Dietary Guidelines Advisory Committee [9], and a recent 2020 Cochrane report [33] all conclude that there are health benefits from replacing saturated fat with polyunsaturated fat.

The findings of this study suggest that students may lack critical knowledge regarding the importance and vital role of the types and amount of fat within the diet, as students overwhelmingly chose non-fat meal dishes as being healthiest. This may be due to a misunderstanding of the roles that saturated and unsaturated fats play in a healthy diet. These results are similar to a global survey which found that less than half of respondents knew that certain dietary fats are essential nutrients [13]. The term “fat” is particularly confusing to the general public, as 90% of those surveyed in a 2009 study reported negative associations with fat [13]. A 2019 study of consumer beliefs about foods and diets found that 54% of respondents from the general public considered a low-fat diet to be healthier when compared to a medium- or high-fat diet [34]. Within the United States, the focus on fat restriction may be changing as a more recent poll of adults found that 58% of those aged 50 or older reported avoiding fat, compared with only 41% of 18- to 29-year-olds [35]. There has also been increased enthusiasm for high-fat diets, such as the ketogenic diet, to aid in weight loss/maintenance and glycemic control. However, these diets lack recommendations about the importance of the type of fat consumed and fail to consider the opportunity cost of the absence of high-fiber, unrefined carbohydrates [32,36,37].

This study found minimal variations between males and females when comparing the healthfulness of no-fat foods versus unsaturated and saturated fat foods. This finding challenges existing research within college student populations which suggests that females avoid high-fat foods and consume more low-fat products than men [38,39]. Avoidance of high-fat foods may be hindered by social stereotypes. In a study of 100 subjects by Barker et al. (1999), a low-fat diet was associated with positive stereotypes, while negative stereotypes were the predominant descriptors of consumers of the high-fat diets [40]. Many of these negative stereotypes surrounding high-fat foods were established early in life due to marketing and are particularly pervasive within females due to social expectations about diet and body image [7,41,42,43]. Further research is warranted to examine how to best overcome engrained false beliefs or perceptions surrounding dietary fat.

Dietary fat is an integral culinary component as it contributes to the texture, flavor, and palatability of foods [4]. Oils and fats also promote the absorption of carotenoids and fat-soluble vitamins [44]. Although adding beneficial, unsaturated fats to meals may slightly increase caloric content, the most up-to-date nutritional science suggests that this is a healthier approach for chronic disease prevention than reducing calories or restricting fat [9,33]. Moderate or even high levels (20–35% of total daily calories) of healthful, beneficial fats (those high in mono- and polyunsaturated fats) are associated with optimal nutrition and maintaining a healthy weight [32,45,46,47]. Emerging research suggests that select amounts of saturated fats may play a role in healthy weight maintenance [10]. Further research is needed to examine how misconceptions about types of dietary fat impact food choices and intake, and how interventions can be designed to target changing dietary behavior to incorporate beneficial fats into a healthy dietary pattern.

Nutrition education should focus on the role of beneficial unsaturated fat within the diet. A study by Yahia et al. (2016) found that increased nutrition knowledge within college students was associated with a reduction in the consumption of saturated and trans-fats [48]. Care should be taken to craft health messaging in ways that the public can understand [49]. Nutrition education materials should stop using low-fat terminology and instead focus on food sources of healthier fats [47,50]. Rather than focusing on percentages of specific types of fat, actionable messaging should focus on total diet quality and food patterns. This shift is supported by the 2015–2020 Dietary Guidelines for Americans and Menus of Change [8,27]. This nutritional education should be taught broadly to students in K-12 schools, included within curricula for dietetics, allied health, and medical students so that they may provide evidence-based recommendations, and incorporated as a key topic in community-based cooking programs.

Research on eating behaviors has long been performed in a scientific laboratory setting, allowing for in-depth understanding of mindset but also separating the research from the point of choice [51]. A strength of this study is the use of dining halls as living laboratories. This approach allows for research at the point of decision: it places nutritional research with the environment where the choice is made and thus approximates real-life conditions. This study utilized the diverse network of schools within the MCURC, which allows for a greater generalizability of the findings.

Several limitations are worth noting. Even though the study’s multisite design helps increase the generalizability of the results, participation in the study was voluntary and all data were obtained from convenience samples at respective MCURC schools. Secondly, only one school (School B) randomized the order of fat options for each meal type. All other schools did not randomize fat options (non-fat option appeared first in the list of the three options, healthy fat appeared second, and saturated fat options appeared third). While unlikely, this non-randomization could have influenced participant’s responses as participants may have quickly selected the same ordered choice for each meal type. There is also a possibility that students were exposed to nutrition educational materials within dining halls earlier in the school year and this may have influenced their responses. Previous research has suggested that race/ethnicity is a determinant in food choices of college students; however, this study did not collect data on race/ethnicity of participants [38]. Data were also not collected on student’s major or field or study, which may have impacted their general knowledge of nutrition. This study did not assess actual consumption of dietary fat, the responses provided by the students provide insight to what their dietary intake might be like [48]. As part of a secondary aim, the fat option was coded into a binary outcome variable with the unsaturated fat response as correct and other responses (no-fat or saturated fat) as incorrect. It should be acknowledged that saturated fat is an imperfect categorization of a group of fatty acids that includes ones with varied effects on serum lipids and perhaps other aspects of health. There is literature that suggests some saturated fat plays a role within a healthy diet [10,52]. Within the context of this study, this binary coding was reflective of the way that fats have been represented educationally and in marketing, thus likely influencing behavior of students.

## 5. Conclusions

Decades of dietary guidance messages recommending restricting total fat in the diet have led to some unintended consequences that have likely been an impediment to the public’s health (i.e., shifting to higher intakes of low quality carbohydrate food choices) [53,54]. Current nutritional science reverses the misconception that we need to limit all fat within the diet. The type of fat is more important than the total amount of fat for chronic disease prevention [55,56]. This study provides a snapshot of the perception of fats with young adults in university and college settings. Nutrition education in these institutions could focus on encouraging dietary intake of healthy, satiating sources of unsaturated fats, which include nuts, seeds, fatty fish, extra virgin olive oil, and avocados. Further research is needed to examine how misconceptions about types of dietary fat impact food choices and intake. Achieving dietary changes to meet the challenge of improving the healthiness of the US diet remains a difficult task. Dietitians, chefs, foodservice industry leaders, and food manufacturers can lead the charge of reshaping consumer attitudes and appetites about healthier dietary fats.

## Figures and Tables

**Table 1:** Faith in fat food item and differing fat options for preparation method.

Food Item	No-Fat Option	Unsaturated Fat Option	Saturated Fat Option
Garden Salad	Lemon Juice Dressing	Olive Oil Dressing	Ranch Dressing
Chicken Breast Entree	Baked with Italian Herbs	Sautéed in Olive Oil	Sautéed in a Lemon Butter Caper Sauce
Oatmeal Cookie Dessert	Prepared with Applesauce	Prepared with Olive Oil	Prepared with Butter

**Table 2:** Characteristics of students participating in the faith in fat study.

Characteristic	All Schools	School A	School B	School C	School D	School E	School F
**Sample (*n*)**	533	52	88	54	81	57	201
**Sex (%)**							
Male	52.2	63.5	54.6	48.2	43.2	64.9	49.3
Female	47.7	34.6	45.5	51.9	56.8	35.1	50.8
Not Listed	0.1	1.9	0	0	0	0	0
**Classification (%)**							
First Year	43.0	42.3	19.3	42.6	93.8	14.0	41.3
Second Year	24.8	25.0	27.3	33.3	2.5	14.0	33.3
Third Year	14.1	11.5	21.6	9.3	2.5	17.5	16.4
Fourth Year	9.9	7.7	25.0	9.3	1.2	7.0	8.5
>Fourth Year	4.3	1.9	5.7	1.9	0	21.1	0.5
Not a Student	3.9	3.9	1.1	3.7	0	26.3	0

**Table 3:** Chi-square of study participant characteristics and fat option at meal type in the faith in fat study.

Meal Type	Sex		Year Classification		School	
	χ2	*p*-Value	χ2	*p*-Value	χ2	*p*-Value
Garden Salad	2.5	0.64	19.6	0.03	44.0	<0.001
Chicken Breast Entree	6.9	0.14	16.4	0.09	51.2	<0.001
Oatmeal Cookie Dessert	12.1	0.02	8.7	0.57	11.0	0.36

## Supplementary Materials

The following are available online at https://www.mdpi.com/2072-6643/12/9/2560/s1, Figure S1: The Principles of Healthy, Sustainable Menus; Table S1: Characteristics of schools participating in the Faith in Fat and Love It or Lose It studies; Table S2: Frequency of fat option choice by sex in the Faith in Fat study; Table S3: Frequency of fat option choice by school in the Faith in Fat study; Table S4: Frequency of fat option choice by year classification in the Faith in Fat study.
