# Supplementary material for: Faith in Fat: A Multisite Examination of University Students’ Perceptions of Fat in the Diet
Source: Nutrients. 2020 Aug 24;12(9):2560. doi: 10.3390/nu12092560 (PMC7551440; doi:10.3390/nu12092560)
Supplement: Supplementary file 1 [file nutrients-12-02560-s001.pdf]

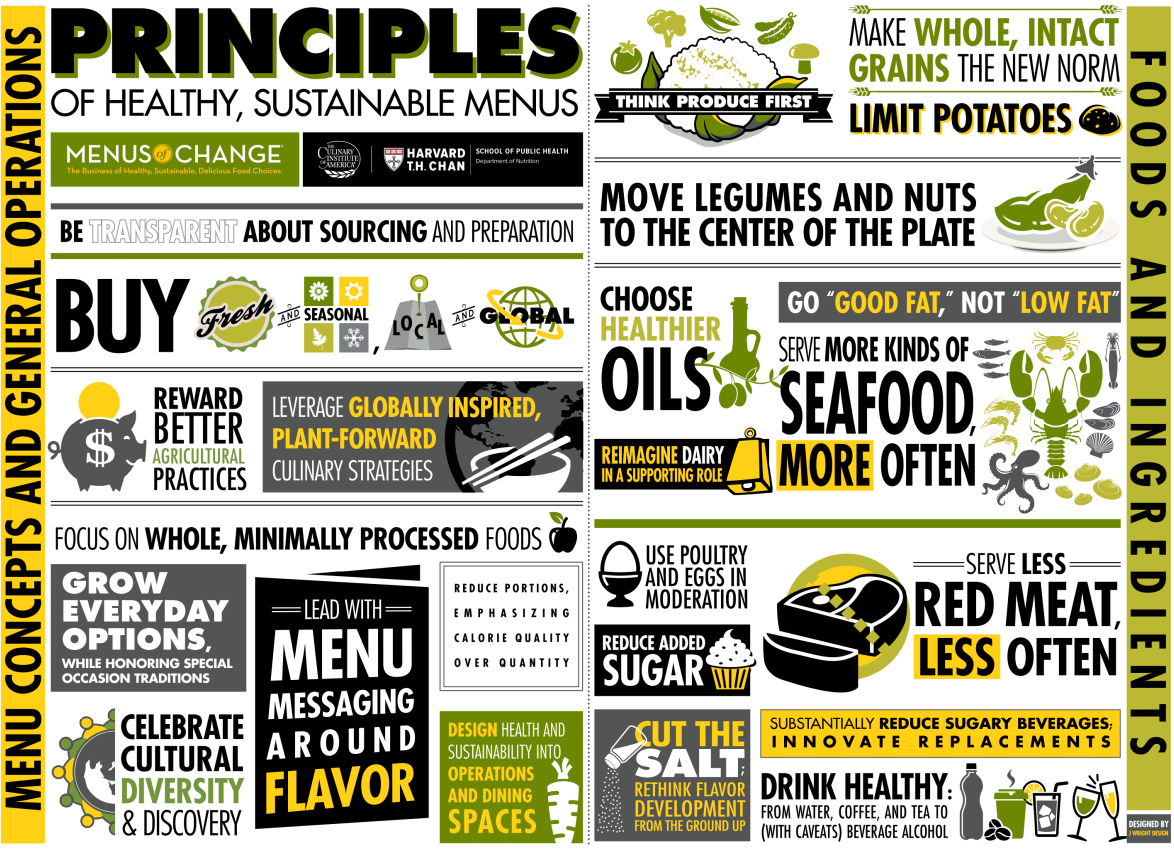

© 2019 The Culinary Institute of America and President and Fellows of Harvard College, as published in the *Menus of Change®* Annual Report. All rights reserved. See the full version of the principles at www.menusofchange.org/principles-resources/moc-principles/

Figure S1: The Principles of Healthy, Sustainable Menus. Developed by the Culinary Institute of America and Harvard T. H. Chan School of Public Health as part of the Menus of Change Initiative, The Principles of Healthy, Sustainable Menus provide unique guidance for the foodservice industry, and bring together findings from nutritional and environmental science perspectives on optimal food choices, trends in consumer preferences, and impacts of projected demographic shifts. The principle "Go 'Good Fat', Not 'Low Fat'" was the focus of this study examining college students' perceptions of health among foods with no-fat relative to foods with different types of fats (unsaturated and saturated) and whether college students can accurately identify foods with healthy (unsaturated) versus unhealthy (saturated) dietary fats. Reproduced with permission from The Culinary Institute of America and President and Fellows of Harvard College.

Table S1. Characteristics of Schools Participating in the Faith in Fat Study

Characteristic	School A	School B	School C	School D	School E	School F
**Undergraduate Population (n)**	31,577	1624	21,384	30,872	7,064	33,677
**Female (%)**	56.7	54.3	45.2	59.3	48.5	50.4
**Race/Ethnicity(%)**						
Asian	28.1	2.2	6.2	28.4	21.6	26.5
Black	3.4	3.7	5.8	2.2	6.7	7.2
Hispanic/Latino	22.2	5.9	5.1	21.4	15.6	13.1
White	26.7	80.4	69.2	25.1	35.6	38.8
International	11.8	11.1	4.7	15.7	9.2	9.0
Other[1]	8.1	6.7	9.1	7.1	11.3	5.4
**Public/Private**	Public	Private	Public	Public	Private	Public
**Population Density**	Urban	Rural	Urban	Suburban	Suburban	Urban
**Geographic Location (in United States)**	West	Northeast	Southeast	West	West	Northeast

[1] Other includes Native Hawaiian or Other Pacific Islander, American Indian, Alaskan Native, or Unknown Ethnicity

Table S2. Frequency of Fat Option Choice by Sex in the Faith in Fat Study[1]

	Sample Size	Salad			Main Entrée			Dessert		
		No Fat	Healthy Fat	Unhealthy Fat	No Fat	Healthy Fat	Unhealthy Fat	No Fat	Healthy Fat	Unhealthy Fat
Male	278	74.5	23.4	2.2	71.9	20.9	7.2	62.6	32.0	5.4
Female	254	79.5	19.3	1.2	78.0	16.9	5.1	74.4	23.2	2.4
Not Listed	1	100	0.0	0.0	0.0	100	0.0	0.0	100	0.0

[1] Numbers reflect percent (%) within sex

Table S3. Frequency of Fat Option Choice by School in the Faith in Fat Study[1]

	Sample Size	Salad			Main Entrée			Dessert		
		No Fat	Healthy Fat	Unhealthy Fat	No Fat	Healthy Fat	Unhealthy Fat	No Fat	Healthy Fat	Unhealthy Fat
School A	52	84.6	15.4	0.0	78.8	19.2	1.9	57.7	36.5	5.8
School B	88	64.8	27.3	8.0	64.8	13.6	21.6	76.1	18.2	5.7
School C	54	70.4	27.8	1.9	77.8	18.5	3.7	70.4	25.9	3.7
School D	81	72.8	27.2	0.0	80.2	17.3	2.5	71.6	24.7	3.7
School E	57	68.4	31.6	0.0	64.9	33.3	1.8	59.6	35.1	5.3
School F	201	86.1	13.4	0.5	77.6	18.4	4.0	67.7	29.9	2.5

[1] Numbers reflect percent (%) within school

Table S4. Frequency of Fat Option Choice by Year Classification in the Faith in Fat Study[1]

	Sample Size	Salad			Main Entrée			Dessert		
		No Fat	Healthy Fat	Unhealthy Fat	No Fat	Healthy Fat	Unhealthy Fat	No Fat	Healthy Fat	Unhealthy Fat
First Year	229	78.6	19.7	1.7	74.7	20.1	5.2	67.2	29.3	3.5
Second Year	132	86.4	12.9	0.8	74.2	17.4	8.3	70.5	26.5	3.0
Third Year	75	70.7	26.7	2.7	82.7	13.3	4.0	70.7	22.7	6.7
Fourth Year	53	67.9	30.2	1.9	69.8	17.0	13.2	67.9	26.4	5.7
> Fourth Year	23	65.2	30.4	4.3	73.9	26.1	0.0	52.2	47.8	0.0
Not a Student	21	57.1	42.9	0.0	61.9	38.1	0.0	71.4	23.8	4.8

[1] Numbers reflect percent (%) within year classification.
